# Allelic Variations in the Human Genes *TMPRSS2* and *CCR5,* and the Resistance to Viral Infection by SARS-CoV-2

**DOI:** 10.3390/ijms23169171

**Published:** 2022-08-15

**Authors:** Girolamo Aurelio Vitello, Concetta Federico, Francesca Bruno, Mirella Vinci, Antonino Musumeci, Alda Ragalmuto, Valentina Sturiale, Desiree Brancato, Francesco Calì, Salvatore Saccone

**Affiliations:** 1 Oasi Research Institute—IRCCS, Via Conte Ruggero 73, 94018 Troina, Italy; 2 Department Biological, Geological and Environmental Sciences, University of Catania, Via Androne 81, 95124 Catania, Italy

**Keywords:** COVID-19, *TMPRSS2* gene, *CCR5* gene, rs35074065, rs12329760, rs333, SARS-CoV-2 first wave infection

## Abstract

During the first wave of COVID-19 infection in Italy, the number of cases and the mortality rates were among the highest compared to the rest of Europe and the world. Several studies demonstrated a severe clinical course of COVID-19 associated with old age, comorbidities, and male gender. However, there are cases of virus infection resistance in subjects living in close contact with infected subjects. Thus, to explain the predisposition to virus infection and to COVID-19 disease progression, we must consider, in addition to the genetic variability of the virus and other environmental or comorbidity conditions, the allelic variants of specific human genes, directly or indirectly related to the life cycle of the virus. Here, we analyzed three human genetic polymorphisms belonging to the TMPRSS2 and CCR5 genes in a sample population from Sicily (Italy) to investigate possible correlations with the resistance to viral infection and/or to COVID-19 disease progression as recently described in other human populations. Our results did not show any correlations of the rs35074065, rs12329760, and rs333 polymorphisms with SARS-CoV-2 infection or with COVID-19 disease severity. Further studies on other human genetic polymorphisms should be performed to identify the major human determinants of SARS-CoV-2 viral resistance.

## 1. Introduction

The infection caused by the severe acute respiratory syndrome coronavirus 2 (SARS-CoV-2) induces coronavirus disease 2019 (COVID-19), which rapidly spread around the world in the last two years. The pandemic originated in China and spread across the world with diversified levels of contagion and mortality in different geographical areas. The causes of this diversification should be found in several factors, such as (1) genetic variability of the virus, (2) genotype of human genes involved in the viral infection process, (3) geographical and climatic features, and (4) high levels of specific environmental pollutants [1]. 

Furthermore, an extremely heterogeneous picture emerged in Italy, with contagion values and mortality of many northern regions that were significantly higher than those of central and southern Italy [2,3]. During the first wave of infection, Italy had one of the highest numbers of SARS-CoV-2 related contagions and one of the highest mortality rates. Across the world, the most severe clinical course of COVID-19 has been associated with old age, comorbidities, and male gender [4,5,6,7]. 

Thus, prognostic markers for the early identification of high-risk subjects, as well as knowledge of the environmental parameters that facilitate virus diffusion, are important to implement specific actions. On the other hand, polymorphisms in specific human genes may have contributed to the high variability in the contagiousness and lethality rates of COVID-19.

The interpretation of the mortality rate (CFR Case-Fatality Rate) of this disease is extremely complex because several factors influence it, including high average age and population density, management policy, the number of performed swabs, and the presence of comorbidities especially in older patients. Several published studies showed that, in Italy, the death rate was positively correlated to the advanced age of patients; however, this cannot alone explain why mortality rates from COVID-19 in Italy are higher than in some countries, like Japan, where the average population age is higher [8,9,10].

A study on the danger of SARS-CoV-2 virus must consider not only the environmental factors and the genetic variability of the virus but also the allelic variants of some specific human genes that can determine a genetic predisposition to the viral infection. Two human genes related to viral infection have been identified, *ACE2* and *TMPRSS2*, whose protein products are directly involved in the viral life cycle. One works as a cell receptor (ACE2) for virus entry into the competent human cells, and the other (TMPRSS2), a serine protease enzyme, allows the priming of the viral spike protein (S). Thus, both proteins are involved in the internalization of the virus into the human cell [7,11,12].

Many papers in the last few years analyzed the role of the Angiotensin-Converting Enzyme 2 (ACE2) and the Type II Transmembrane Serine Protease (TMPRSS2) in the mechanism of the coronavirus infection. The pivotal role of ACE2 as a receptor of viral S protein for virus entry into the host cells was demonstrated as was the role of serin protease TMPRSS2 in activating the spike protein and facilitating viral entry [13,14]. Studies of the human proteins involved in the virus cell cycle contribute to a better understanding of the variability of SARS-CoV-2 infection susceptibility, of the severity of disease symptoms, and outcomes in the mortality rate in the involved populations; however, there is still much to uncover [15,16,17].

Polymorphisms in the *TMPRSS2* gene were analyzed regarding their involvement in SARS-CoV-2 infection, and some common variants seem to modulate *TMPRSS2* expression, allowing a mild-to-moderate effect in infection susceptibility [4,18]. Genetic variants related to reduced *TMPRSS2* expression may confer less individual susceptibility to infection with a better disease outcome. Indeed, greater frequency of alleles with high expression levels of *TMPRSS2* was related to higher COVID-19 prevalence and mortality rates in Europe and the Americas, contrary to the lower prevalence of the disease and mortality observed in Southeast Asia, where alleles characterized by a low level of expression of *TMPRSS2* gene were described [19].

In addition to the TMPRSS2 gene, other studies showed the involvement of CC chemokine receptor 5 (CCR5) in genetic susceptibility of SARS-CoV-2 infection. The CCR5-∆32 I/D polymorphism was detected as a promising candidate to predict the severity of SARS-CoV-2 infection. The specific deletion seems to be protective against COVID-19 disease [20]. As CCR5 is not a receptor recognized by SARS-CoV-2, the most likely explanation for the protective effect of the ∆32 allele in COVID-19 is an attenuated inflammatory response among the CCR5-deletion carriers [21,22].

A relevant fact to be considered concerns the variability of the viral infection, and of the clinical symptoms of the disease, observed in cohabiting subjects (families, hospital communities, and retirement homes for the elderly) in which only one person was infected by SARS-CoV-2 and developed the disease, while the other members of the group showed no virus or disease symptoms. Thus, if we consider families or, in general, communities, the specific genotype of each cohabiting subject could have a role in the different responses to viral infection. Here, taking into consideration the results previously described for some polymorphisms of *TMPRSS2* and *CCR5* genes in different human populations (see above), we analyzed three polymorphisms (rs35074065, rs12329760, and rs333) to detect the presence of an allele or a genotype that could confer resistance to viral infection and/or result in less severe symptoms of COVID-19 disease.

## 2. Results

### 2.1. Subjects

A total of 102 subjects were recruited for the present study (Table 1). The subjects belong to families in which some members were infected in the first pandemic wave (March and April 2020), when the original strain of SARS-CoV-2 circulated in Italy. Their genomic DNA samples were collected in 2021, from May to December. None of the subjects analyzed were vaccinated at the time of the infection, as vaccines were not yet available. 

The subjects belong to two different groups living in the same place, with several of them infected by the SARS-CoV-2 virus and others not. One group is composed of subjects who were hospitalized in the intellectual disability ward of the Oasi M. Santissima Scientific Hospitalization and Care Institutes (IRCCS; Troina, Italy), and the other group consists of subjects belonging to 10 different families, each composed of three to five cohabiting members, where at least one member was infected by SARS-CoV-2 virus and the other members not.

### 2.2. TMPRSS2 Gene

Two different SNPs belonging to *TMPRSS2* gene were analyzed, rs35074065 and rs12329760. The former consists of a deletion of the C nucleotide (delC) and has an allele frequency in the European population of 17.4%. The second is a nucleotide substitution (C > T) with the T allele in the European population showing a frequency of 22.0% (dbSNP database at www.ncbi.nlm.nih.gov/snp/ accessed on 21 April 2021).

**Figure 1:**
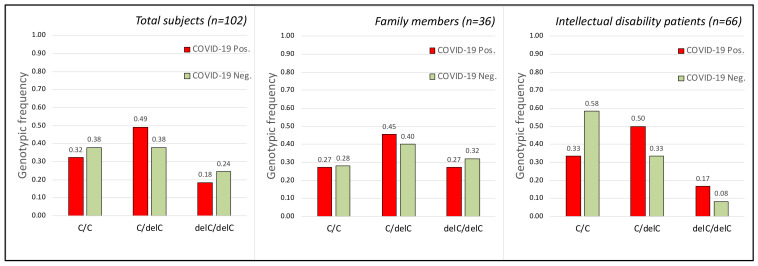
Frequency of the three genotypes obtained by the C and delC alleles of the rs35074065 SNP polymorphism (TMPRSS2 gene) in the analyzed groups. (**Left**) Genotype frequency in the total subjects (Chi-square Test for 3 *×* 2: Chi = 0.019 *p* = 0.991 df = 2; Sample size = 102; power = 0.132740). (**Middle**) Genotype frequency in the subjects belonging to the analyzed families with cohabiting members (Chi-square Test for 3 *×* 2: Chi = 0.1119 *p* = 0.9456 df = 2). (**Right**) Genotype frequency in the subjects belonging to the intellectual disability patients cohabiting in the Oasi M. Santissima IRCCS (Troina, Italy) (Chi-square Test for 3 *×* 2: Chi = 2.65 *p* = 0.265 df = 2). Each group was subdivided into infected (COVID-19 Pos.) and non-infected (COVID-19 Neg.) groups. No statistically significant correlation was obtained between SARS-CoV-2 infection and the rs35074065 SNP polymorphism.

**Figure 2:**
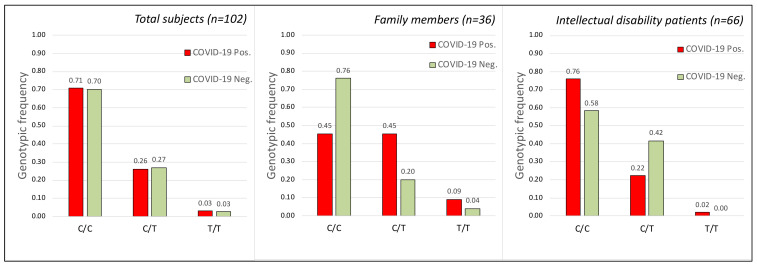
Frequency of the three genotypes obtained by the C and T alleles of the rs12329760 SNP polymorphism (TMPRSS2 gene) in the analyzed groups. (**Left**) Genotype frequency in the total subjects (Chi-square Test for 3 *×* 2: Chi = 1.282 *p* = 0.527 df = 2; Sample size = 102; power = 0.0922357). (**Middle**) Genotype frequency in the subjects belonging to the analyzed families with cohabiting members (Chi-square Test for 3 *×* 2: Chi = 3.207 *p* = 0.2011 df = 2). (**Right**) Genotype frequency in the subjects belonging to the intellectual disability patients cohabiting in the Oasi M. Santissima IRCCS (Troina, Italy). Each group was subdivided into infected (COVID-19 Pos.) and non-infected (COVID-19 Neg.) groups. (Chi-square Test for 3 *×* 2: Chi = 2.0812 *p* = 0.353 df = 2). No statistically significant correlation was obtained between SARS-CoV-2 infection and the rs12329760 SNP polymorphism.

The genotypic analysis of these two polymorphisms does not show significant correlation (see Figure 1 and Figure 2) between susceptibility of SARS-CoV-2 virus infection and the alleles detected in rs35074065 (Figure 1) and rs12329760 (Figure 2) SNPs in the human *TMPRSS2* gene.

### 2.3. CCR5 Gene

**Figure 3:**
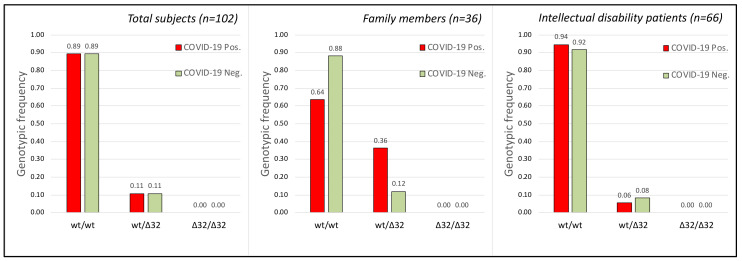
Frequency of the three genotypes obtained by the wild-type (wt) and ∆32 alleles of the rs333 SNP polymorphism (CCR5 gene) in the analyzed groups. (**Left**) Genotype frequency in the total subjects (Chi-square Test for 2 *×* 2: Chi = 0.00004 *p* = 0.995 df = 1; Sample size = 102; power = 0.15933). (**Middle**) Genotype frequency in the subjects belonging to the analyzed families with cohabiting members (Chi-square Test for 2 *×* 2: Chi = 2.8948 *p* = 0.0888; df = 1). (**Right**) Genotype frequency in the subjects belonging to the intellectual disability patients cohabiting in the Oasi M. Santissima IRCCS (Troina, Italy). Each group was subdivided into infected (COVID-19 Pos.) and non-infected (COVID-19 Neg.) groups. Chi-square Test for 2 *×* 2: Chi= 0.1331 *p* = 0.7153; df = 1). No statistically significant correlation was obtained between SARS-CoV-2 infection and the rs333 SNP polymorphism.

The rs333 polymorphism, consisting of a deletion of 32 bp in the *CCR5* gene, was analyzed. This allele, ∆32, shows a frequency in the European population of 11.0% (dbSNP database at www.ncbi.nlm.nih.gov/snp/ accessed on 21 April 2021). The genotypic analysis of the polymorphism showed no significant correlation (see Figure 3) with susceptibility to SARS-CoV-2 virus infection (Figure 3).

### 2.4. TMPRSS2, CCR5 Gene Polymorphisms and COVID-19 Disease Severity

The three polymorphisms rs35074065, rs12329760, and rs333 were analyzed in relation to the genotypes observed in intellectual disability patients with respect to the severity of the disease. We distinguished three levels of disease severity in addition to the asymptomatic cases (pauci-symptomatic, symptomatic, and severe) as follows:

*Asymptomatic:*

Patients have no symptoms.

*Pauci-symptomatic:*

1. Headache, rhinitis, pharyngitis, fatigue, mild cough, muscle aches.
2. Fever < 37.5 °C.
3. Peripheral SpO_2_ saturation >95.

*Symptomatic:*

1. Convulsive cough.
2. Fever < 37.5 °C.
3. Mild dyspnea.
4. Peripheral SpO_2_ saturation >90–<95.

*Severe:*

1. Severe dyspnea.
2. Fever > 37.5 °C.
3. Marked Desaturation SpO_2_ < 90; P/F < 300.

**Figure 4:**
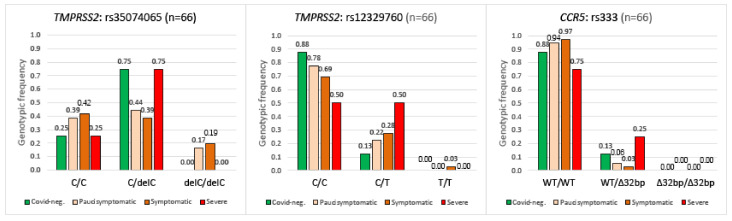
Genotype distribution of the rs35074065, rs12329760, and rs333 polymorphisms in the intellectual disability patients cohabiting in the Oasi M. Santissima IRCCS (Troina, Italy) sub-divided by severity of COVID-19 disease. rs35074065: Chi-square = 5.482, *p* = 0.483, df = 6; rs12329760: Chi-square = 3.077, *p* = 0.799, df = 6; and rs333: Chi-square = 3.792, *p* = 0.284, df = 3. No statistically significant correlation was obtained between disease severity and the three polymorphisms analyzed.

No significant correlation (see Figure 4) was found between disease severity and the genotypes of the three polymorphisms analyzed (Figure 4).

Finally, when comparing infected (COVID-19 positive) and non-infected (COVID-19 negative) individuals (females and males separately), genotypic frequencies of the loci rs333, rs35074065, and rs12329760 failed to show statistical significance (Table 2).

### 2.5. Genotype Concordance between Infected and Non Infected Members in Cohabiting Subjects

The main input of the work was the verification of whether a specific genotype, among those considered by us, determines, in the carrier, a resistance to the viral infection or, conversely, a higher susceptibility to it, and the rationale to use individuals who live in the same “environment” (a home) allowed the elimination of all environmental variables that could interfere in the infection process. Thus, we verified if there were families with infected subjects cohabiting with healthy members who had different genotypes. 

This type of information (Table 3) clearly showed that there are no genotypes among those analyzed that could have a direct correlation with the COVID-19 disease or with resistance to SARS-CoV-2 infection. In fact, except for the T/T genotype of the rs12329760 polymorphism, which was, however, very rare in the population analyzed, all the other genotypes showed a random distribution between individuals who were positive and negative for viral infection, in accordance with the statistical analyses on the general population or divided into the two family and hospitalized groups (Figure 1, Figure 2 and Figure 3). As an example, the genotype C/C of the SNP rs12329760 is present in infected and non-infected members in five families (Table 3).

## 3. Discussion

Environmental factors, such as temperature, humidity, and air pollutants, have been correlated with the incidence of new cases, mortality rates, and the spread of the virus in different Italian regions [1]. In fact, PM_10_ and PM_2.5_ airborne particulate matters at high concentrations can influence the circulation of the virus in the air acting as a carrier and can exert a boost action, thus, stimulating the high diffusion of the epidemic [23]. Despite the widespread diffusion of the virus and independent of the mode of action (by interpersonal infection or by environmental spread), some subjects showed no viral infection even though they cohabited with infected subjects, some of whom died from COVID-19. 

Thus, genetic variation among cohabiting subjects should be considered to explain the different responses to SARS-CoV-2 infection. Thus far, it is still unclear why the severity of COVID-19 disease is so highly variable between infected individuals: indeed, some do not develop any symptoms (asymptomatic), while others develop mild ones (pauci-symptomatic). In other cases, individuals may present with a respiratory syndrome that may result in intensive care. Here, we analyzed three polymorphisms of the human *TMPRSS2* and *CCR5* genes to highlight possible associations between alleles/genotypes and resistance/sensitivity to viral infection or different symptoms related to COVID-19 disease. 

Subjects from families and a hospital community in Sicily (Italy) with different levels of susceptibility or responses to SARS-CoV-2 infection were included in the study. Cohabitation (in a community or in a household) represents one of the main risk factors for COVID-19 infection. Indeed, exposure to the same viral strain may result in either infection sensitivity or resistance or in highly variable levels of disease severity. Thus, when we take into consideration households or communities, the genetic variability of each household or community member should play a role in the differing responses to viral infection.

It was previously described that polymorphisms of the *CCR5* gene may have an impact on susceptibility to viral infection with COVID-19 [5]. Similarly, a significantly higher CCR5-Δ32 frequency was observed in symptomatic SARS-CoV-2 positive patients compared to COVID-19 asymptomatic subjects. Moreover, these researchers found that CCR5 deletion may predict the severity of SARS-CoV-2 infection [4]. Thus, countries with a higher CCR5-Δ32 frequency showed the highest viral infection and COVID-19 mortality rates [24,25]. In our work, the statistical data are supportive of the fact that CCR5-Δ32 mutation is not a risk factor for SARS-CoV-2 infection nor for the disease course (see Figure 3 and Figure 4), at least in the Sicilian population we analyzed. This is similar to what was observed in Germany [26].

Several studies on the genetic contribution of *TMPRSS2* polymorphisms (rs35074065, rs12329760) to individual susceptibility to viral infection [27] either confirmed [14,27,28,29] or disconfirmed [30,31,32] this correlation. Our results did not indicate any correlation between viral infection and the analyzed polymorphisms.

Moreover, we analyzed the three polymorphisms, rs35074065 and rs12329760 in the *TMPRSS2* gene and rs333 in the *CCR5* gene, in a cohort of patients with intellectual disability residing in rehabilitation departments of the Research Institute “IRCCS Oasi Maria SS”, based in Troina, Italy. Patients were divided into subgroups matched by COVID-19 disease severity: asymptomatic, pauci-symptomatic, symptomatic, and severe, and in this case no statistically significant differences were found between groups (Figure 4) even when we considered only female patients (data not shown). 

No statistical significance was obtained when grouping asymptomatic and pauci-symptomatic vs. symptomatic and severe, or when using allele frequencies for statistical analysis (data not shown). No correlation was found between age and disease severity (r = 0.19896181, *p*-value = 0.139439) even when considering only female (90.7%) patients (r = 0.211702, *p*-value = 0.147545). Similarly, when comparing infected (COVID-19 positive) and non-infected (COVID-19 negative) individuals, genotypic or allele frequencies of the loci rs333, rs35074065, and rs12329760 failed to reach statistical significance (Figure 1, Figure 2 and Figure 3) even when considering females and males separately (Table 2).

Although the “sample size” analysis had shown our population as being appropriate for the statistical result obtained (sample size about 102, see Figure 1, Figure 2 and Figure 3), certainly, a larger sample would have given more robustness “power” to the work (power= 0.132740, 0.0922357, and 0.15933 for SNPs rs35074065, rs12329760, and rs333, respectively). Unfortunately, to our knowledge, there are not many families that show both members infected by SARS-CoV-2 and other members who were not; furthermore, with the advent of vaccinations, it is no longer possible to obtain new samples, as, during correlation analysis, the protective effect of the vaccine would overlap with gene polymorphisms. 

Finally, the difference between the observed genotype frequencies of the three polymorphisms in the two compared groups (infected and not infected, Figure 1, Figure 2 and Figure 3) is very low—predominantly 1%. The chi-square tests obtained are indeed strongly indicative of this negative correlation, statistically confirmed by the high “*p*” value obtained (*p* = 0.991, 0.527, and 0.995, for SNPs rs35074065, rs12329760, and rs333, respectively). In summary, our data highlight that the three analyzed human polymorphisms (rs35074065, rs12329760, and rs333) in the samples from Sicily (Italy) are indicative that no correlation exists with the increased risk factor for SARS-CoV-2 infections, nor are they correlated to disease course. 

Thus, these polymorphisms cannot be the cause of the different responses to viral infection detected among subjects cohabiting in the same house or in the same community building. Further studies on other human polymorphisms are necessary to identify the major determinants of SARS-CoV-2 viral resistance observed in these subjects, and work is in progress to analyze samples from different countries, which can include vaccinated and non-vaccinated subjects, to show whether the three polymorphisms here studied or elsewhere identified can be relevant in the vaccinated individuals.

## 4. Materials and Methods

### 4.1. Subjects

Biological materials, and data were collected from May to December 2021; however, infection of the analyzed subjects occurred in the first pandemic wave (Mar/May 2020). The present study was conducted on a cohort of 102 subjects from Sicily (Italy) with a personal or familial history related to the first wave of COVID-19 infection (Supplementary Table S1). We analyzed people living in the same place, belonging to the same family, or hospitalized in the same hospital ward, some of whom were infected by the SARS-CoV-2 virus and showed symptoms of the disease (65 cases), and other cohabitants not showing infection or symptoms (37 cases).

The collected biological samples consisted of peripheral blood or exfoliative buccal cells. All procedures performed in the study were in accordance with the ethical standards of the institutional and/or national research committee and with the 1964 Helsinki declaration and its later amendments or comparable ethical standards. Signed informed consent forms were obtained from the analyzed subjects. This study was approved by the local ethics committee “Comitato Etico IRCCS Sicilia—Oasi Maria SS”, approval code: 2021/05/04/CE-IRCCS-OASI/43 as of 5 May 2021.

### 4.2. Nasopharyngeal Swabs for SARS-CoV-2 Detection

We used diagnostic Vita PCR SARS-CoV-2 platforms (Menarini Diagnostics, Wokingham, UK, and Credo Diagnostics, Singapore) following a protocol previously described [33] or the COVID-19 CE-IVD RT-PCR by Applied Biosystems test. This tool detects two SARS-CoV-2 RNA target sequences, one in the (specific) virus nucleocapsid (N) gene region and the other in the conserved region of SARS-like viruses (including the SARS-CoV-2, SARS-CoV, and SARS-like bat coronaviruses), respectively. Thus, the genotype of SARS-CoV-2 was not detected, as the diagnostic tool was not specific for virus strain identification.

### 4.3. DNA Preparation and Analysis

Genomic DNA was prepared from whole peripheral blood or buccal exfoliated cells using the MagCore Compact Automated Nucleic Acid Extractor (RBC Bioscience, New Taipei City, Taiwan). DNA samples were obtained following the standard manufacturer’s protocols suggested for each type of biological material.

PCR amplifications were performed using the Applied Biosystems Veriti Thermal Cycler (Thermo Fisher Scientific, Waltham, MA, USA). The amplification parameters were 94 °C/30 s, 55 °C/30 s, and 72 °C/30 s per cycle for 35 cycles. The primers used to amplify the DNA segments were:

- *TMPRSS2* gene, polymorphism rs12329760:
  - for: 5′-TCTGCTGTCTGTTACTGTCACT-3′,
  - rev 5′-ACTCATGGATAATCCTCCCTC-3′.
- *TMPRSS2* gene, polymorphism rs35074065:
  - for: 5′-GGGCCCCCAAAGTAACCAATGGA-3′,
  - rev 5′-ATGGACCATTGAGCCAGTGCTTATGT-3′.
- *CCR5* gene, polymorphism rs333:
  - for: 5′-[6FAM] CTGTGTTTGCGTCTCTCCCA-3′,
  - rev 5′-CCTCTTCTTCTCATTTCGACAC-3′.

**Figure 5:**
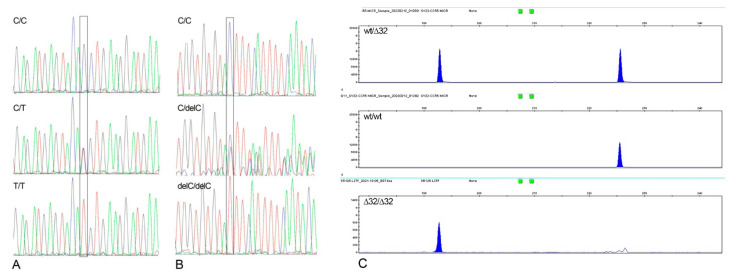
Visualization of the genotypes for the rs12329760, rs35074065, and rs333 polymorphisms. (**A**) Sanger sequencing electropherograms for the rs12329760 polymorphism of the *TMPRSS2* gene. (**B**) Sanger sequencing electropherograms for the rs35074065 polymorphism of the *TMPRSS2* gene. (**C**) Gene Mapper visualization of the rs333 polymorphism of the *CCR5* gene. All the genotypes are indicated.

The genotypes of the polymorphisms rs12329760, and rs35074065 were obtained by sequencing the amplified DNA segments (Figure 5A,B). ExoSAP purified samples were used before direct sequencing of PCR products. Sequencing was performed on a SeqStudio using a BigDye Terminator Cycle Sequencing Ready Reaction Kit (Applied Biosystems, Waltham, MA, USA).

The genotypes of the polymorphisms rs333 (CCR5-∆32) were obtained by capillary electrophoresis with SeqStudio and GeneMapper 4.0 software (Figure 5C). Forward primer for ∆32bp deletion (rs333) of *CCR5* gene was modified on the 5′ end by the addition of a dye label (6FAM). After PCR amplification, 1 μL of the products was diluted in 12 μL of deionized formamide and 1 μL of GeneScan 350 Rox (molecular weight DNA marker). The DNA was denatured at 95 °C for 3 min and loaded on an SeqStudio Genetic Analyser (Applied Biosystems) for amplicon length determination.

### 4.4. Statistical Analysis

The experimental design evaluated:

(1) The differences between four COVID-19 categories (asymptomatic, pauci-symptomatic, symptomatic, and severe) and the genotypes of each of the three loci analyzed in 66 subjects with intellectual disability using a 4 × 3 contingency table.
(2) The genotypes and allele frequencies between infected and non-infected patients using a 3 × 2 contingency table McNemar test. *p* values less than 0.05 were considered statistically significant. If the total N for a 2 × 2 chi-square table was less than about 40, the Yates continuity correction was used to compensate for deviations from the theoretical probability distribution. The calculation of the Pearson Correlation Coefficient was conducted using online website software: https://www.socscistatistics.com, accessed on 5 May 2022. Sample size: the statistical appropriateness of the chi-squared tests used in this study was assessed *a posteriori* by first calculating the effect size and then calculating the corresponding sample size required with α = 0.05 (www.statskingdom.com/34test_power_chi2.html, accessed on 5 May 2022).

## Figures and Tables

**Table 1:** Subjects enrolled in this study.

	With Intellectual Disability ^(1)^		Family Members ^(2)^	
	COVID-19 Infected	COVID-19 Non-Infected	COVID-19 Infected	COVID-19 Non-Infected
N°	54	12	11	25
Mean age (±SD)	42.42 (±11.38)	40.49 (±17.05)	36.78 (±18.11)	32.64 (±17.11)
Female/Male	49/5	10/2	5/6	15/10
(Female %)	(90.7%)	(83.3%)	(45.4%)	(60.0%)

^(1)^ Hospitalized and living in the same hospital ward. ^(2)^ Cohabiting subjects belonging to 10 different families.

**Table 2:** Statistical analysis between infected (COVID-19 positive) and non-infected (COVID-19 negatives) females and males.

Infected vs. Non-Infected	TMPRSS2 rs35074065	TMPRSS2 rs12329760	CCR5 rs333
Females	Chi = 2.157 *p* = 0.34	Chi = 1.33 *p* = 0.51	Chi = 2.22 *p* = 0.14
Males	Chi = 0.71 *p* = 0.70	Chi = 2.4 *p* = 0.12	Chi = 0.15 *p* = 0.696

**Table 3:** Distribution of the genotypes in the analyzed families.

	*SNP rs12329760*			*SNP rs35074065*			*SNP rs333*		
*SARS-CoV-2*	*C*/*C*	*C*/*T*	*T*/*T*	*C*/*C*	*C*/*delC*	*delC*/*delC*	*wt*/*wt*	*wt*/*∆32*	*∆32*/*∆32*
Positive members	5	5	1	3	5	3	7	4 *	0
Negative members	19	5	1	7	10	8	22	3	0
Family numbers with positive and negative members ^(1)^	5	3	0	1	3	2	7	2	0

^(1)^ Number of families where the same genotype was detected in positive and negative members. * Two positive members are in the same family.

## Data Availability

All data were in the Table S1.

## Supplementary Materials

The following supporting information can be downloaded at: https://www.mdpi.com/article/10.3390/ijms23169171/s1.
